# Pointer Defect Detection Based on Transfer Learning and Improved Cascade-RCNN

**DOI:** 10.3390/s20174939

**Published:** 2020-09-01

**Authors:** Weidong Zhao, Hancheng Huang, Dan Li, Feng Chen, Wei Cheng

**Affiliations:** School of Electrical Information and Engineering, Anhui University of Technology, Ma’anshan 243032, China; zwd720819@163.com (W.Z.); hhcair@163.com (H.H.); hellochenf@163.com (F.C.); cwzdtbs@163.com (W.C.)

**Keywords:** pointer, defect detection, transfer learning, deformable convolution, online hard example mining

## Abstract

To meet the practical needs of detecting various defects on the pointer surface and solve the difficulty of detecting some defects on the pointer surface, this paper proposes a transfer learning and improved Cascade-RCNN deep neural network (TICNET) algorithm for detecting pointer defects. Firstly, the convolutional layers of ResNet-50 are reconstructed by deformable convolution, which enhances the learning of pointer surface defects by feature extraction network. Furthermore, the problems of missing detection caused by internal differences and weak features are effectively solved. Secondly, the idea of online hard example mining (OHEM) is used to improve the Cascade-RCNN detection network, which achieve accurate classification of defects. Finally, based on the fact that common pointer defect dataset and pointer defect dataset established in this paper have the same low-level visual characteristics. The network is pre-trained on the common defect dataset, and weights are transferred to the defect dataset established in this paper, which reduces the training difficulty caused by too few data. The experimental results show that the proposed method achieves a 0.933 detection rate and a 0.873 mean average precision when the threshold of intersection over union is 0.5, and it realizes high precision detection of pointer surface defects.

## 1. Introduction

With the popularization of automated production [1,2,3,4,5], detection technology based on machine vision [6,7,8,9,10,11,12,13] has greatly promoted the development of the automobile manufacturing industry. Compared with traditional manual inspection, it has the advantages of high efficiency and high accuracy. There are many defects that will inevitably occur in the production process of automotive dashboard pointers. Some examples are surface pollution, hair-like defects, hot stamping paper folds, hot stamping paper damage and needle light leakage. These defects will directly affect the stability of the automotive instrument system. Therefore, it is of great significance to study the detection methods of pointer surface defects.

The traditional defect detection method of auto parts based on machine learning is mainly to extract the defect features by manual means, and then send the extracted features to support vector machine (SVM), AdaBoost and other classifiers for classification and recognition. Liu et al. [14] proposed an improved OTSU algorithm to extract the appearance defect features of the image and realized the detection of sleeve defects in the automotive steering gear. Li [15] studied the pit defects on the surface of turbine shell parts and obtained the region of interest by using an accurate boundary extraction algorithm based on ellipse fitting. The morphological features are extracted by image segmentation technology and then sent to the support vector machine for classification. Zhang et al. [16] improved the Canney operator through an adaptive filtering method and combined it with the watershed algorithm to obtain different enhanced image features. Then, the support vector machine classifier was trained according to the image features of the surface defects on the automotive injection molded threaded parts. Meng et al. [17] used HALCON algorithm for the processing of the automotive hose image. Moreover, they proposed a method to reduce misjudgment and identify the surface defects of the hose. Tandiya et al. [18] developed a semi-specular surface defect detection system, and designed specific filters to eliminate spurious defects due to edges and acute curvature changes. This system was able to detect various defects on the car bumper and had good robustness. However, the methods based on machine vision described above have poor ability to mine data and low detection accuracy.

In recent years, the favorable conditions have been created for the accurate and rapid detection with the development and application of deep learning. Du et al. [19] modified the Faster-RCNN algorithm by using feature pyramid network (FPN), used different data enhancement methods to make up for the lack of the images in the dataset and improved the defect detection accuracy of X-ray image on automotive castings. Zhao et al. [20] segmented the collected car wheel images and enhanced the image contrast and defect characteristics through image processing technology. Then, the convolutional neural network (CNN) was used to extract the characteristics of the defects, which realized accurate detection of wheel hub surface defects. Zhang et al. [21] constructed the thickness cloud map and Gaussian curvature cloud map of automotive cover parts and built a model based on Faster-RCNN to realize the detection of wrinkle and crack defects on automotive cover parts. Wu et al. [22] created a blade defect dataset and proposed an end-to-end framework based on residue learning. The data equalization operation was integrated into the process of detection and extensive experimental validation was conducted. The results demonstrate that the framework can achieve a better defect detection result. Qu et al. [23] proposed the PartsNet to realize the detection of automotive engine precision parts. PartsNet consists of a pixel-wise segmentation network and a feature refining network. The basic features of parts defects were learned by segmentation network, and then several typical methods of refinements were transformed into convolutional manners. The detection results show that the model established has good adaptability and portability. To sum up, the auto parts detection methods based on deep learning can extract rich features of images, which perform better than the traditional methods.

**Figure 1:**
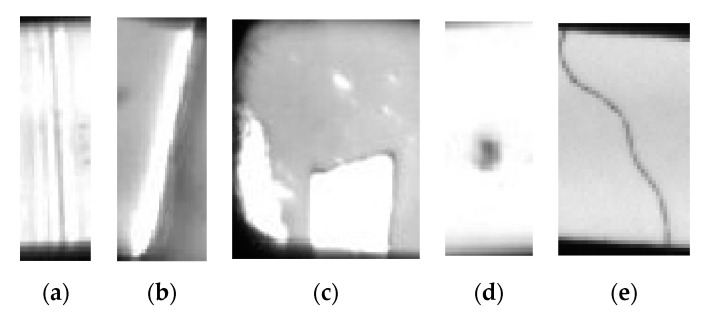
Examples of pointer surface defects: (**a**) hot stamping paper folds; (**b**) hot stamping paper damage; (**c**) needle leakage; (**d**) stains; and (**e**) hair-like defects.

**Figure 2:**
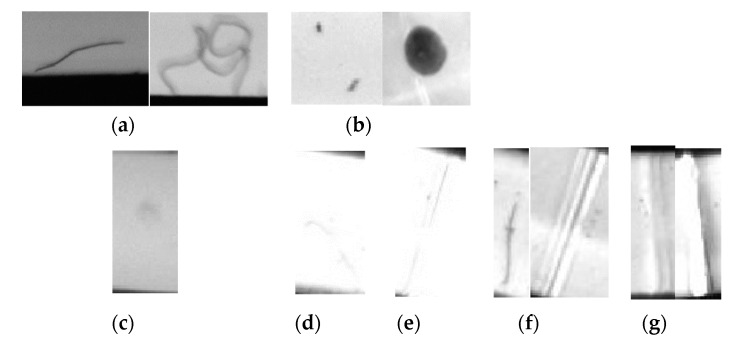
(**a**) Hair-like defects have different appearances; (**b**) stains have different appearances; (**c**) weak feature of stains; (**d**) weak feature of hair-like defects; (**e**) weak feature of hot stamping paper folds; (**f**) similarity exists in defects of hair-like defects and hot stamping paper folds; and (**g**) similarity exists in defects of hot stamping paper folds and hot stamping paper damage.

Based on the five types of defects proposed by automotive dashboard pointer manufacturers, this paper constructs a pointer surface defect dataset (including hot stamping paper folds, hot stamping paper damage, needle leakage, stains and hair-like defects), as shown in Figure 1. By comparing the characteristics of various types of defects, the difficulties of the inspection task are analyzed: (1) Differences exist in some defects which are of the same type, and the same types of defects have different appearances. For example, some hair-like defects are shaped in curved line and some in clusters (Figure 2a) and some stain-type defects are small and some are large (Figure 2b). (2) Some defects have weak features, such as stains (Figure 2c), hair-like (Figure 2d) and hot stamping paper folds (Figure 2e). The visual effect is very weak and cannot be quickly distinguished by the naked eye. (3) Similarities exist in defects of different types. For example, defects in hair-like matter are similar to those in hot stamping paper folds (Figure 2f) and defects in hot stamping paper folds are similar to those in hot stamping paper damage (Figure 2g). (4) The sample size of this dataset is too small, and there are only 372 processed images. Aiming at the above difficulties, this paper proposes a transfer learning and improved Cascade-RCNN deep neural network (TICNET) algorithm for detecting pointer defects, which can detect five types of defects on the pointer accurately.

## 2. Theories and Methods

### 2.1. Overview of Cascade-RCNN

The application of deep neural network to image processing has achieved satisfying results. Furthermore, convolutional neural network (CNN) has strong capability of feature extraction, and it plays an important role in the field of object detection [24]. The output of a convolutional layer can be described as:

(1) $$z(u,v)=\sum_{i=-\infty }^{\infty }\sum_{j=-\infty }^{\infty }x_{i,j}*p_{u-i,v-j}$$

where z(u,v) is the output, $x_{i,j}$ represents the input images and p represents the convolution kernels with different sizes. The object detection tasks based on CNN are usually divided into two types: one-stage and two-stage tasks. One stage [25,26] represents that the category probability and position coordinates of the object are returned directly. On the other hand, the process of object detection can also be completed with a convolutional neural network in two stages [27]. It means that the region proposal network is trained firstly and then the object detection network will be trained.

**Figure 3:**
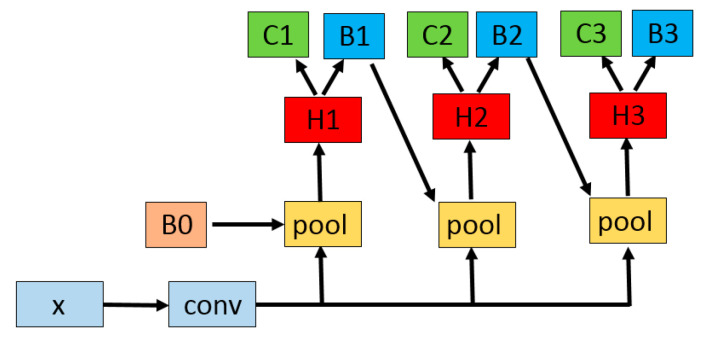
This is the structure of Cascade-RCNN.

Cascade-RCNN [28,29] expands the classical two-stage structure to a multi-stage structure. High quality positive sample training is performed at each stage by setting different IoU (Intersection over Union) thresholds. Hence, the accuracy of bounding box detected and adjusted will be improved. The structure of Cascade-RCNN is shown in Figure 3, where *x* is the input; *conv* is the convolutional layer of the basic network; B0 is the proposal frame generated by the region proposal network; *pool* is the pooling layer; H1, H2 and H3 are the detection network; C1, C2 and C3 are classifiers; and B1, B2 and B3 are bounding box regressors.

### 2.2. Basic Network ResNet-50 Reconstructed by Deformable Convolution

Some defects on the surface of the dashboard pointers are different within the class, and features of some defects are weak. Therefore, it is difficult for the convolutional neural network to completely “remember” the diversity of the pointer surface defects through the dataset. Because conventional convolutional neural network is limited to a fixed geometric structure during modeling and the sampling position of the convolution unit on the input image remains unchanged each time, not only is the feature loss serious, but also the fitting ability of loss function becomes weak, and the overall performance of the network degrades. To solve the above problems, this paper introduces deformable convolution [30,31,32] to reconstruct the convolutional layers of the basic network ResNet-50 [33,34,35]. The conventional convolution structure is defined as follows:

(2) $$y(p_{0})=\sum_{p_{n}\in R}w(p_{n})x(p_{0}+p_{n})$$

where $p_{n}$ is the offset of each point relative to each point on the receptive field after sampling, usually an integer; *R* is the sampling grid; and $w(p_{n})$ is the sampling weight. The deformable convolution is to add an offset $\Delta p_{n}$ to each point, which is obtained by another convolution and is usually a non-integer value. The deformable convolution structure is defined as follows:

(3) $$y(p_{0})=\sum_{p_{n}\in R}w(p_{n})x(p_{0}+p_{n}+\Delta p_{n})$$

The value $x(p_{0}+p_{n}+\Delta p_{n})$ in the above formula is not an integer and does not correspond to the actual point on the feature map. This requires the derivation of discontinuous position variables, so bilinear interpolation is used:

(4) $$x(p)=\sum_{q}G(q,p)x(q)=\sum_{q}g(q_{x},p_{x})g(q_{y},p_{y})x(q)$$

where x(q) represents the values of points at all integer positions on the feature map and $x(p)=x(p_{0}+p_{n}+\Delta p_{n})$ represents the values of points at non-integer positions after adding the offset.

**Figure 4:**
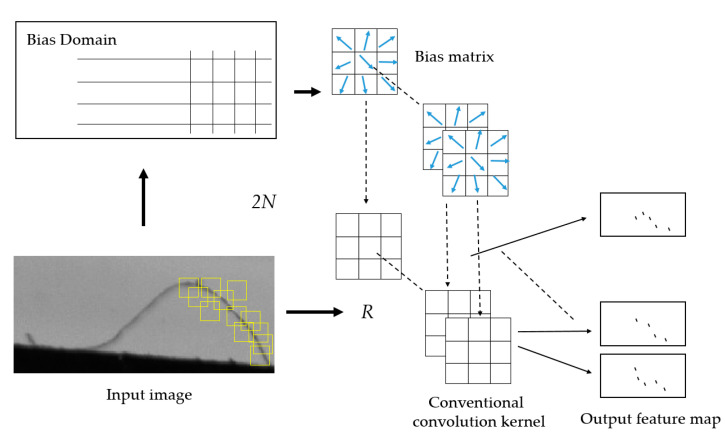
This is an example of deformable convolution calculation process.

**Figure 5:**
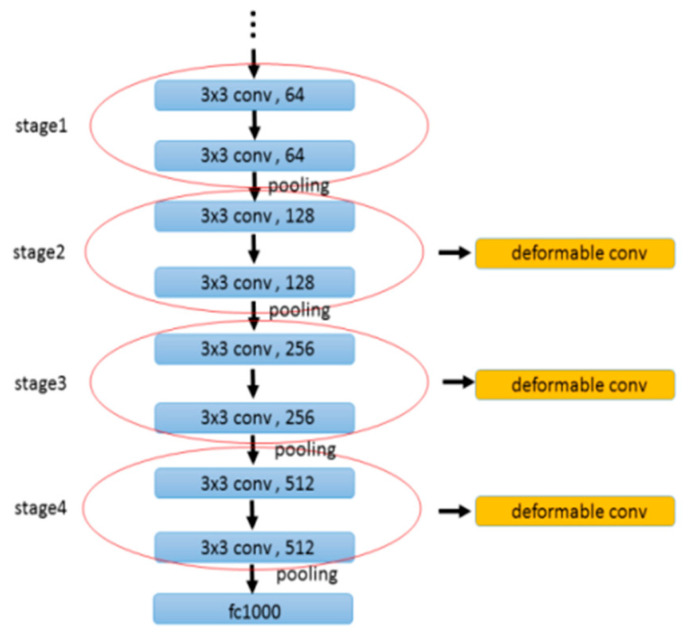
The reconstruction of the ResNet-50.

Deformable convolution expands the grid of conventional convolution into an offset matrix, which contains the offset $\left\{\Delta p_{n}|n=1,\;\ldots ,\;N\right\}$, where N=|R|. Taking hair-like defects as an example, the deformable convolution calculation process is shown in Figure 4, where the size of the offset domain is consistent with the size of the input image. The offset domain is learned by a convolutional layer with the same size as the input image to obtain a two-dimensional offset matrix. If the number of two-dimensional offset matrixes is *N*, the channel dimension will be 2*N*. Because of the offset matrix, not only the sampling positions of the convolution are diversified, but also the sampling points pointed to by the offset domain have a stronger tendency to the target, and there is more output characteristic information. The basic network of Cascade-RCNN is ResNet-50, which has four stages. According to the difference within class and weaker characteristics of the defects on the surface of the pointer, convolutional layers of Stages 2–4 (as shown in Figure 5) are reconstructed to improve the ability to extract features.

### 2.3. OHEM Integrated into the Detection Network

For training samples, they can be divided into simple samples and difficult samples. Simple samples are those that are easy to correctly classify, and difficult samples are those that are misclassified. Simple samples have little significance on model training, while difficult samples can better guide the direction of model optimization due to their higher loss value and greater influence on classification and detection results. Some types of defects belong to difficult samples because they have similar characteristics, and making full use of them can improve the robustness of network and the ability to recognize targets.

**Figure 6:**
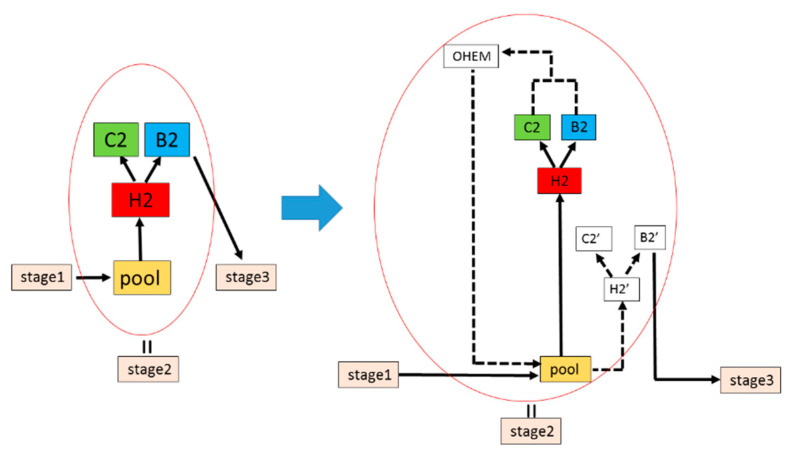
Cascade-RCNN’s detection network is improved by online hard example mining.

Based on the idea of online hard example mining [36,37], this paper improves Cascade-RCNN’s detection network. As shown in Figure 6, taking Stage 2 as an example (the same as Stages 1 and 3), the original detection network H2, C2 and B2 is duplicated as H2′, C2′ and B2′ branches. The H2, C2 and B2 branches are denoted as *L1*, while H2′, C2′ and B2′ branches are denoted as *L2*. *L1* and *L2* share the network’s parameters. *L1* is an only readable forward detection network, while *L2* is a readable and writable standard detection network. First, the candidate regions generated by the region proposal network generate corresponding feature maps through the pooling layer. The feature maps input to *L1* for forward transfer, and the loss values are calculated. Then, the OHEM module sorts the loss values of all candidate areas to screen out some difficult samples with large loss values. Finally, these difficult samples are input into *L2* through the pooling layer for forward calculation and back propagation.

### 2.4. Transfer Learning

The essence of transfer learning [38,39,40] is transferring the features learned from the source domain [41,42,43] to the target dataset. Compared with traditional machine learning methods that require retraining every time, transfer learning can improve the efficiency of training and the adaptability of the model [44]. The lower-level convolutional layers in the convolutional neural network can learn the detailed semantic features such as edges and colors. Furthermore, such more complex features such as different shapes and other combinatorial characteristics can be extracted by the higher-level convolutional layers. 

**Figure 7:**
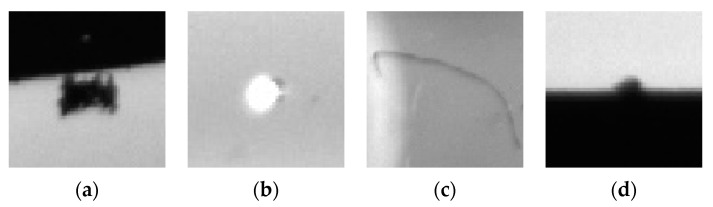
There are four kinds of defects in the common pointer surface defect dataset: (**a**) stain; (**b**) bright spot; (**c**) filament; and (**d**) edge gap.

**Figure 8:**
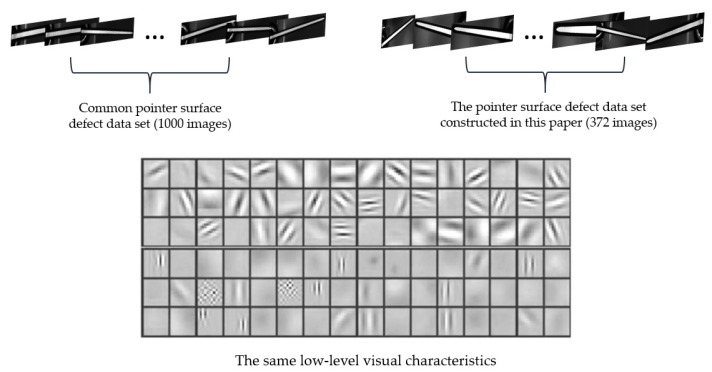
The common pointer defect dataset and the pointer defect dataset constructed in this paper have the same low-level visual characteristics.

The common pointer surface defect dataset comes from pointer images collected by a company. It consists of 1000 images, covering stains, bright spots, filaments and edge gaps (as shown in Figure 7). Figure 8 presents that the common pointer defect dataset and the pointer defect dataset constructed in this paper have the same low-level visual characteristics. Consequently, it will transfer the same features learned previously to the current task when the lower-level convolutional layers are frozen.

**Figure 9:**
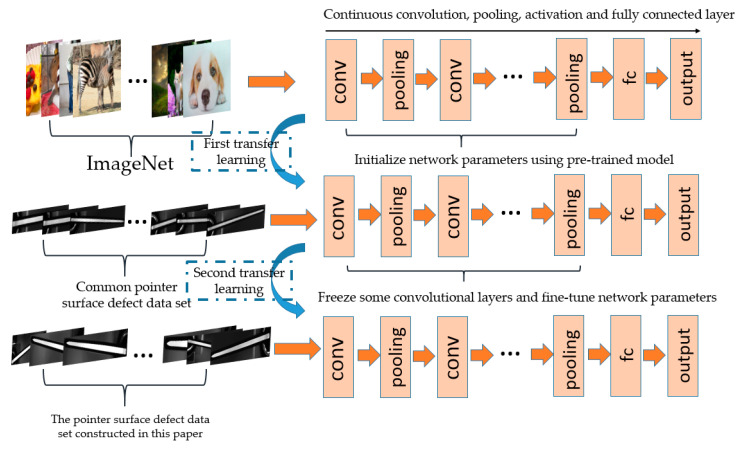
This is the process of transfer learning.

This paper adopts the method of transfer learning to solve the problem that the current dataset is only 372 images, and the process is shown as Figure 9. Firstly, it uses a transfer learning to initialize the network parameters trained on the ImageNet large-scale dataset. Secondly, it tunes the network parameters through the images in the common pointer defect dataset with relatively sufficient data, and a network is trained to recognize the surface defects of common pointer. Finally, the lower-level convolutional layers are frozen, and the secondary transfer learning is performed. Moreover, the higher-level convolutional layers are retrained to learn the complex features. To sum up, the similarity between the source domain and the target domain has been greatly improved. The same low-level visual characteristics are effectively utilized and the complex features are relearned, which alleviates the problem of too few samples.

## 3. TICNET: Proposed Pointer Defect Detection Algorithm

### 3.1. Network Structure

**Figure 10:**
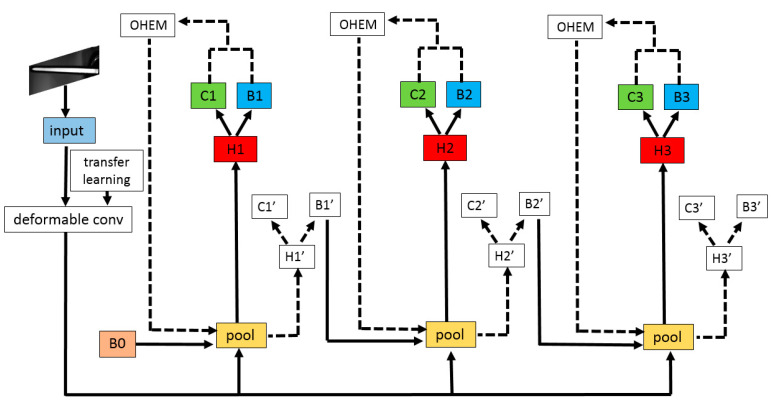
The network structure of TICNET.

The network structure of TICNET is shown in Figure 10. It uses deformable convolution to reconstruct the basic network which can improve the ability to extract feature, and improves the three branches of Cascade-RCNN. The original H1-C1-B1, H2-C2-B2 and H3-C3-B3 branches are copied into H1′-C1′-B1′ branch, H2′-C2′-B2′ branch and H3′-C3′-B3′ branch. OHEM modules are connected in parallel beside the original three branches, which can improve the ability to distinguish similar samples. Combined with the transfer learning theory, the network parameters trained on the common defect dataset are used for initialization. Stages 1–2 of ResNet-50 are frozen and prevented from updating parameters. The parameters at other levels of the network are fine-tuned to make the model converge quickly and overcome the drawback of too few data.

### 3.2. Loss Function

TICNET is formed by cascading multiple sub-networks; each sub-network has a separate detector. The results of detectors are used as the input of the next stage, and the final stage combines the results to output. The cascading relationship of the entire network is shown in Formula (5):

(5) $$f(x,b)=f_{T}of_{T-1}o\ldots of(x,b)$$

where *T* represents the number of cascades, which is 3. Regressor $f_{t}$ at each level correspond to training samples $b_{t}$ at each level. $b_{t}$ is derived from the results of the initial distribution $b_{1}$ after all previous branch outputs, instead of directly using initial distribution of the region proposal network to train $f_{t}$. The sample distribution ratio $b_{t}$ obtained after each level of regression is a bit more accurate than $b_{t-1}$, and the IoU threshold will be increased a bit after each level of regression. This can solve the problem of imbalance between positive and negative samples at each stage.

The loss function of TICNET consists of the bounding box regression loss function and the classification loss function. The regression loss function of the bounding box is defined as follows:

(6) $$T_{loc}\left[F\right]=\frac{1}{N_{j}}\sum_{i=1}^{N_{i}}\sum_{j=1}^{N_{j}}L_{loc}^{j}(F_{T}(x_{i},b_{i}),g_{i})$$

The coordinates and enclosing area of the bounding box can be expressed with $b=(b_{x},b_{y},b_{w},b_{h})$. $b_{x}$ and $b_{y}$ are the coordinates of the center point. $b_{w}$ and $b_{h}$ are the width and height of the bounding box. The real bounding box can be expressed with $g=(g_{x},g_{y},g_{w},g_{h})$, where $g_{x}$ and $g_{y}$ are the coordinates of the center point of the real bounding box and $g_{w}$ and $g_{h}$ are the width and height of the real bounding box. The bounding box makes the IoU between the bounding box and the real bounding box as large as possible through the regression function. *j* represents the *j*th detector of the cascade layer, $N_{j}$ is the number of detectors at this level and $N_{i}$ is the number of predicted samples. $F_{T}(x_{i},b_{i})$ is the bounding box input by the detector and *T* is the level of the detection network. The definition of the $L_{loc}$ function is as follows:

(7) $$L_{loc}(t,o)=\sum_{i\in \left\{x,y,w,h\right\}}{smooth}_{L1}(o_{i}-t_{i})$$

where $o=(o_{x}+o_{y}+o_{w}+o_{h})$ is the frame result output by the regression box, *t* is the vector of real coordinates and ${smooth}_{L1}(x)$ is defined as follows:

(8) $${smooth}_{L1}(x)=\left\{ \begin{array}{l} 0.5x^{2},if\;|x|<1\\ |x|-0.5,otherwise \end{array}\right.$$

The classification loss function is defined as follows:

(9) $$R_{cls}\left[h\right]=\sum_{i=1}^{n}L_{cls}(h(x_{i}),y_{i})$$

where *L_cls_* is the classical cross entropy loss function. The bounding box calculates the label value of the category by weighted average:

(10) $$b^{T}=\frac{1}{N_{i}}\sum_{i=1}^{N_{i}}F_{T-1}^{i}(x^{T-1},b^{T-1})$$

Therefore, the loss function of the *T* level is:

(11) $$L(x^{T},g)=R_{cls}(h_{T}(x^{T}),y^{T})+\beta \left[y^{T}\geq 1\right]T_{loc}(F_{T}(x^{T},b^{T}),g)$$

where $h_{T}$ is the classification result output by the *T*th class classifier; $F_{T}$ is the mean value of the regression result output by the *T*th regressor; β is the weight coefficient, which serves to balance the normalized weight of the classification loss and the frame regression loss; and $y^{T}$ is the label of $x^{T}$ under a given threshold and defined as follows:

(12) $$y=\left\{ \begin{array}{l} g_{y},\mathrm{IoU}(x,g)\geq u\\ 0,\;otherwise \end{array}\right.$$

## 4. Results and Discussion

### 4.1. Preparatory Work

**Figure 11:**
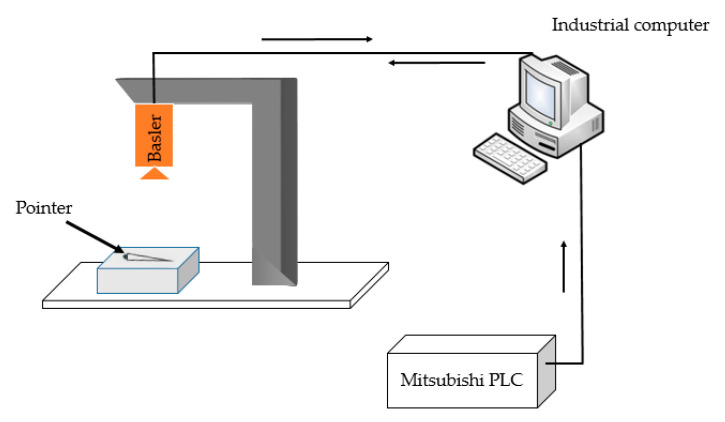
This is the overall detection framework. The role of the industrial computer is to receive the real-time image of the camera and detect defects, while the role of the PLC is to send detection signals.

The 372 images used in the experiment were all cropped from the pictures taken by Basler industrial camera, with a pixel size of approximately 2800 × 300 pixel. We made a dataset of pointer surface defects referring to the format of standard dataset VOC2007 (hot stamping paper folds are marked as fold, hot stamping paper damage are marked as rupture, needle leakage are marked as light leaking, hair-like defects are marked as hair and stains are marked as taint). We randomly selected 300 images to form the training set, and the remaining 72 images formed the testing set. During training, the images were randomly flipped with a probability of 0.5 for data enhancement. The experimental platform is shown in Table 1 and the overall detection framework is shown in Figure 11. 

### 4.2. Detection Results

The TICNET network model was used to detect five types of defects on the pointer surface, and we counted the average precision (AP) and average confidence (AVGConf) when the threshold of intersection over union (IoU) was 0.5 for each category. The definition of Precision and AP are as follows:

(13) $$\mathrm{Precision}=\frac{TP}{TP+FP}$$

(14) $$\mathrm{AP}=\frac{1}{11}\sum_{r\in \{0,0.1,0.2,\ldots ,1\}}\rho_{interp(r)}$$

where *TP* represents the positive samples detected correctly and *FP* represents the negative samples detected incorrectly. $\rho_{interp(r)}$ can be expressed as:

(15) $$\rho_{interp(r)}=\underset{\tilde{r}:\tilde{r}\geq r}{max}\rho (\tilde{r})$$

where $\rho (\tilde{r})$ is the precision at recall $\tilde{r}$, and Recall is defined as:

(16) $$\mathrm{Recall}=\frac{TP}{TP+FN}$$

where *FN* represents the positive samples detected incorrectly. Therefore, AP was obtained by interpolating the precision at the eleven equally spaced levels [0, 0.1, 0.2, …, 1] and taking the maximum precision whose recall value is greater than *r*. The detective confidence (*Conf*) is defined as:

(17) $$Conf=P_{i}({class}_{j}|Object)$$

It represents the probability that the defect belongs to category *j*, when there are objects in the current detection box *i*. For each category of defects, we defined the average confidence (*AVGConf*) as:

(18) $$AVGConf_{j}=\frac{1}{n}\sum_{i=1}^{n}{Conf}_{i}$$

where *n* is the sum of detected boxes that contain category *j*.

**Figure 12:**
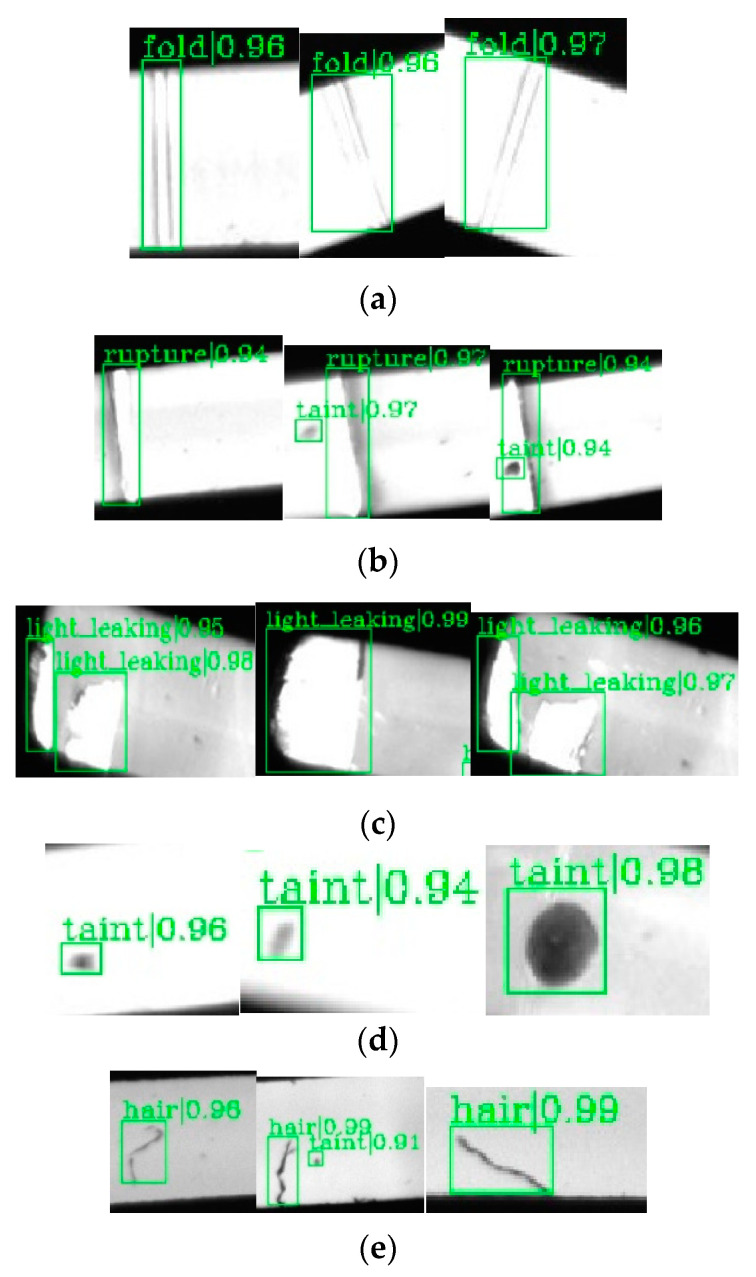
The results of defect detection: (**a**) hot stamping paper folds; (**b**) hot stamping paper damage; (**c**) needle leakage; (**d**) stains; and (**e**) hair-like defects.

The evaluation results on the testing set are shown in Table 2, and randomly selected detection results are shown in Figure 12. Table 2 shows that the defects detection has high average precision. Although the detective precision of hot stamping paper damage is lowest, the value of AP_0.5_ is still up to 0.852 on the testing set. Figure 12 and Table 2 show that each category has a *AVGConf* of ≥0.9 and all detected boxes have high precision. The manufacturer has a demand that both detective precision and confidence are above 85%, thus we conclude that the detection performance of TICNET is satisfactory.

### 4.3. Ablation Study

#### 4.3.1. Ablation Study for TICNET

To quantitatively prove the effectiveness of methods such as deformable convolution, online hard example mining and transfer learning, the method of controlling variables was used to conduct comparative experiments on the following four schemes. The experiments of the four schemes were all trained for 30 rounds under the same hardware configuration. The schemes were as follows:

- Scheme 1: The proposed algorithm does not use deformable convolution to improve the basic network and still uses conventional convolution to build ResNet-50.
- Scheme 2: The proposed algorithm does not use online hard example mining, and the detection network still uses Stages 1–3 of Cascade-RCNN.
- Scheme 3: Secondary transfer learning is abandoned. The pre-trained weights of ImageNet are used to train the TICNET, instead of using the model that can recognize common pointer surface defects as the pre-trained model.
- Scheme 4: The complete model of TICNET is used.

In this research, the mean average precision (mAP), accuracy (ACC) and detection rate (DR) were also used as the evaluation indices. The mean average precision (mAP) is defined as:

(19) $$\mathrm{mAP}=\frac{1}{n_{j}}\sum_{j=1}^{n_{j}}{\mathrm{AP}}_{j}$$

where *n_j_* is the total number of categories and here is 5 and ${\mathrm{AP}}_{j}$ is the average precision of each category. In addition, mAP_0.5_ and mAP_0.7_, respectively, represent the mAP when the thresholds of IoU are 0.5 and 0.7. 

The accuracy (ACC) is defined as:

(20) $$\mathrm{ACC}=\frac{TP+TN}{TP+FP+TN+FN}$$

where *TN* represents the negative samples detected correctly. The detection rate (DR) is defined as:

(21) $$\mathrm{DR}=\frac{1}{n_{k}}\sum_{k=1}^{n_{k}}{\mathrm{IoU}}_{k}$$

where $n_{k}$ is the sum of detected boxes, ${\mathrm{IoU}}_{k}$ is the intersection over union of the box *k* and the definition of IoU can be expressed as:

(22) $$\mathrm{IoU}=\frac{area(B_{P}\cap B_{gt})}{area(B_{P}\cup B_{gt})}$$

where $B_{P}$ is a predicted bounding box and $B_{gt}$ is the ground truth bounding box.

Table 3 presents the experimental data of Schemes 1–4. It is shown that the mAP_0.5_ and mAP_0.7_ have, respectively, declined 9.9% and 9.0% under the circumstance that Scheme 1 is used, which are remarkable decreases compared with Schemes 2 and 3. As a reason, the deformable convolution can extract richer features. It can be found that the detection rate increases 10.2% on condition that the OHEM is used by comparing Schemes 2 and 4. The reason is that the indistinguishable samples in the training process are fully utilized, thereby improving the recognition ability of the network. The metrics of mAP_0.5_ and mAP_0.7_ have, respectively, declined 5.4% and 6.6% when the pre-trained weights of ImageNet are used to train the TICNET (Schemes 3 and 4), and thus we conclude that the secondary transfer learning has a positive impact on the network and can also improve the overall performance of the network.

**Figure 13:**
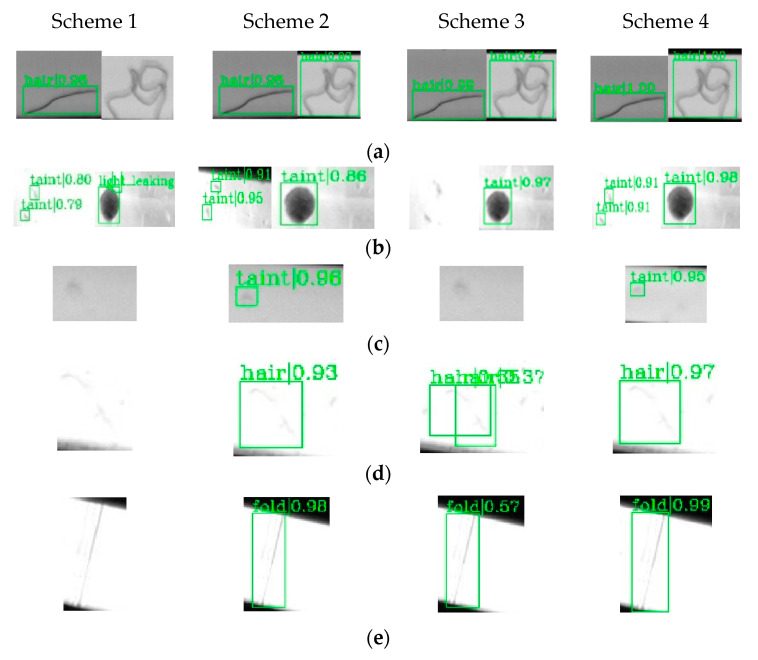

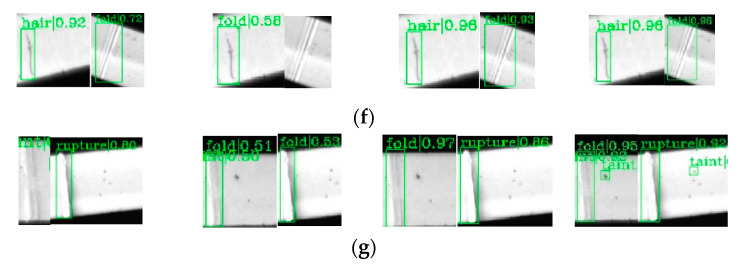
The detection results of four schemes for different situations: (**a**) hair-like defects have different appearances; (**b**) stains have different appearances; (**c**) weak feature of stains; (**d**) weak feature of hair-like defects; (**e**) weak feature of hot stamping paper folds; (**f**) similarity exists in defects of hair-like defects and hot stamping paper folds; and (**g**) similarity exists in defects of hot stamping paper folds and hot stamping paper damage.

To verify the effects of deformable convolution, online hard example mining and transfer learning, the models of Schemes 1–4 were used to verify the seven situations in Figure 2, respectively, and the results are shown in Figure 13. 

Through the comparison and analysis with the complete TICNET model (Scheme 4), it can be seen that the clustered hair and defects with weak features are not detected when the deformable convolution is not used to reconstruct ResNet-50 (Scheme 1). Moreover, large stains are mistakenly detected as needle light leakage and some of the detected defects have low confidence. As a reason, the network’s ability to extract deep features is weak when deformable convolution is not used. 

It is found that the hair-like defect is mistakenly detected as hot stamping paper folds, and hot stamping paper damage is falsely detected as hot stamping paper folds when OHEM is not used to improve the inspection network (Scheme 2). The reason is that these samples with high loss values are not effectively used, which reduces the discriminative ability of the network. It can also be found that the stains with weak features are not detected when transfer learning is not used. Moreover, the hair-like defect with weak feature has lower frame accuracy, and the confidence of some detected defects are lower. As an explanation, the random initialization of network parameters makes it difficult to achieve optimal results under the same number of training rounds.

#### 4.3.2. Ablation Study for Transfer Learning

The changes of variables in transfer learning will affect the results, and we attempted to find an optimal set of variables to better fit this detection task. Hence, a comparative experiment was carried out, and the results are shown in Table 4. 

In this experiment, the independent variables included transfer learning strategy, backbone and frozen stages. On the other hand, the dependent variables included parameters ACC and inference time/group. Among them, the transfer learning strategy consists of different combinations and the frozen stages represents the frozen stages of ResNet during tuning. Besides, backbone is the basic network and we chose ResNet-34, ResNet-50 and ResNet-101 for comparison. We define the parameters of the common convolutional layers as:

(23) $${\mathrm{Params}}_{conv}=K_{h}\times K_{w}\times C_{in}\times C_{out}+C_{out}$$

where $K_{h}\times K_{w}$ is the kernel size, $C_{in}$ is the number of input channels and $C_{out}$ is the number of output channels. The parameters of the fully connected layers can be defined as:

(24) $${\mathrm{Params}}_{fc}=N_{in}\times N_{out}+N_{out}$$

where $N_{in}$ represents that the input has $N_{in}$ nodes and $N_{out}$ represents that the output has $N_{out}$ nodes. Therefore, the number of parameters (Params) is the sum of the all convolutional and fully connected layers’ parameters. The last column of Table 4 shows the inference time and each group contained 10 images.

In Table 4, it can be seen that the number of parameters is only related to frozen stages and backbone, but not to the transfer learning strategy. It is clear that the increase of ResNet depth dramatically resulted in the increase of parameters and inference time. Moreover, ACC was also improved significantly if we replaced ResNet-34 with ResNet-50. On the other hand, although the increase of ResNet depth may achieve an improvement of ACC, ResNet-101 was not able to improve the ACC with a lower cost of inference time. Hence, it can be concluded that ResNet-50 is a better choice than ResNet-34 or ResNet-101.

The number of frozen stages affects the parameters and inference time as well. In other words, the more stages are not frozen, the more parameters and inference time are added. However, the accuracy will be lost if too many or too few stages are frozen. By comparing Methods 4 and 7–9, it is found that the accuracy obtained by Methods 7–9 is not as high as that obtained by Method 4.

The comparative results show that ACC was influenced by the transfer learning strategy as well. Methods 1–4 indicate that ACC is the worst when we train the TICNET from scratch. Furthermore, when the pre-trained weights of Pascal VOC were used in the first transfer stage, rather than the pre-trained weights of ImageNet, it was found that ACC declined from 0.906 to 0.884. Thus, it can be inferred that the transfer learning can improve the performance of TICNET, and the pre-trained weights of ImageNet is more suitable for our task. 

### 4.4. Comparison of Different Detection Algorithms

To prove the superiority of the proposed algorithm, several classic detection algorithms were compared with TICNET in the same experimental environment. We still used mAP_0.5_, mAP_0.7_, ACC and DR as the evaluation indicators. Comparison algorithms include SSD [46], Retinanet [47], RFCN [48], Faster-RCNN [49], Cascade-RCNN, SSD *, RFCN * and Cascade-RCNN *. Among them, SSD *, RFCN * and Cascade-RCNN * represent that we trained the model from scratch. The comparative results are shown in Table 5.

Table 5 demonstrates that the detection results of SSD are the worst, and the mAP_0.5_ and mAP_0.7_ are both below 0.7. Moreover, although the detection results of Retinanet are improved compared with those of SSD, the effect is still not satisfactory. This is due to the fact that both SSD and Retinanet belong to the one-stage detection networks. In other words, the original image is scaled by one-stage detection networks, which made it difficult to detect small targets because of the receptive field. Notably, the detection indicators of RFCN and Faster-RCNN are all over 0.8, which is a big step forward from SSD and Retinanet. The reason is that both RFCN and Faster-RCNN are two-stage detection networks, and the region proposal network plays an important role in detecting small targets. There are many anchors eventually learned in the one-stage network, but most anchors are adverse to the learning process. Anchors in this part greatly affect the whole network and lower the overall accuracy. In addition, the selective search strategy of region proposal network can filter out the redundant anchors. Although fewer anchors are eventually learned from the two-stage network, there will not be too many anchors unfavorable to online learning. They may affect the overall accuracy rate, but it is certainly not as severe as that of one-stage, so the accuracy rate is higher than that of one-stage. Nevertheless, the overall performance of RFCN and Faster-RCNN is still not as good as TICNET.

It is found that the detection indicators of Cascade-RCNN are all lower than those of the two-stage detection networks and TICNET. As an explanation, the defects of the detection task in this paper are complex and some defects have similar characteristics. The feature extraction ability of the basic network is weak, and the samples that have been mistakenly detected are not properly utilized. Therefore, it is concluded that the Cascade-RCNN without any improvements is not suitable for our task. On the other hand, whether the weight is transferred or not also affects the detection indicators. The comparison results show that the migration of weights will effectively improve the performance.

Table 6 illustrates the comparison statistics of algorithms on the different defects. SSD and Faster-RCNN are chosen as the comparison algorithms for TICNET, and the average confidence (AVGConf) is used as evaluation indicator. 

The aforementioned table indicates that the average confidence of each type rises by at least 12.5% and 2.7%, respectively, when compared with SSD and Faster-RCNN. Consequently, it is inferred that the method proposed in this article is more sensitive to the defects on the pointer surface, and the network is more robust due to the improved methods proposed in this article. To summarize, the TICNET has the best performance in our detection task, which is superior to other classical detection algorithms. This is of great significance to ensure the quality of pointers in the production.

## 5. Conclusions

Visual inspection of industrial precision parts is a research hotspot in the computer vision field today, and inspection methods based on machine vision have been applied in industry. Based on the study of pointer surface defects, this paper proposes a transfer learning and improved Cascade-RCNN deep neural network for the problems of intra-class differences, weak features, similarities in different classes and small sample size. The proposed algorithm uses deformable convolution to enhance the feature extraction ability of the feature extraction network. Drawing on the idea of online hard example mining, the detection branches containing the OHEM module are merged into the original detection branches, which effectively improves the network’s ability to distinguish complex samples. The transfer learning theory is used in this detection task to solve the problem of lack of samples and imbalance of data. Comparative experiment results show that the proposed algorithm can accurately detect various defects on the pointer surface. The mAP_0.5_ is 0.873, ACC is 0.906 and DR is 0.933, which are better than the current classical detection algorithms. The TICNET network model has achieved good inspection results in the task of detecting defects on pointer surface, and it is expected to be widely used in the field of industrial vision inspection in the future.

## Figures and Tables

**Table 1:** Hardware and software parameters of the experimental platform.

Name	Parameter
Camera	Basler acA5472-5gm, and the resolution is 5472 × 3648
CPU	Intel Core i7-6800 @ 3.4 GHz
Memory	32 GB
GPU	Dual-channels NVIDIA GTX1080Ti
Operating system	Linux Ubuntu 16.04
Deep Learning Framework	PyTorch 1.3.1 [45]
Language	Python 3.7.5

**Table 2:** The evaluation results on the testing set.

Name of the Defects	AP_0.5_^1^	AVGConf ^2^
Hot Stamping Paper Folds	0.864	0.969
Hot Stamping Paper Damage	0.852	0.957
Needle Leakage	0.877	0.974
Stains	0.911	0.971
Hair-Like Defects	0.861	0.989

^1^ AP_0.5_ is the average precision when the threshold of intersection over union is 0.5. ^2^ AVGConf is the average confidence.

**Table 3:** Comparative results for the proposed algorithm.

Schemes	mAP_0.5_^1^	mAP_0.7_^2^	ACC ^3^	DR ^4^
Scheme 1 ^5^	0.774	0.792	0.825	0.843
Scheme 2 ^6^	0.825	0.804	0.871	0.831
Scheme 3 ^7^	0.819	0.796	0.864	0.907
Scheme 4 ^8^	0.873	0.862	0.906	0.933

^1^ mAP_0.5_ is the mean average precision when the threshold of intersection over union is 0.5. ^2^ mAP_0.7_ is the mean average precision when the threshold of intersection over union is 0.7. ^3^ ACC is the accuracy. ^4^ DR is the detection rate. ^5^ The deformable convolution is abandoned. ^6^ The OHEM is abandoned. ^7^ The secondary transfer learning is abandoned. ^8^ The complete model of TICNET is used.

**Table 4:** Comparative results for different methods.

Method Number	Transfer Learning Strategy	Backbone	Frozen Stages	Params(M) ^1^	ACC ^2^	Inference Time/Group (s)
1	- ^3^	ResNet-50	Stages 1–2	67.72	0.719	1.569
2	Weights of Pascal VOC ^4^	ResNet-50	Stages 1–2	67.72	0.865	1.576
3	Weights of Pascal VOC + Weights of CPD ^5^	ResNet-50	Stages 1–2	67.72	0.884	1.580
4	Weights of ImageNet + Weights of CPD (Ours)	ResNet-50	Stages 1–2	67.72	0.906	1.574
5	Weights of ImageNet + Weights of CPD	ResNet-34	Stages 1–2	33.01	0.817	1.133
6	Weights of ImageNet + Weights of CPD	ResNet-101	Stages 1–2	86.71	0.908	2.010
7	Weights of ImageNet + Weights of CPD	ResNet-50	Stage 1	68.94	0.899	1.589
8	Weights of ImageNet + Weights of CPD	ResNet-50	Stages 1–3	60.62	0.862	1.492
9	Weights of ImageNet + Weights of CPD	ResNet-50	-	69.17	0.870	1.588

^1^ Params(M) is the number of model parameters. ^2^ ACC is the accuracy and it defined as formula (20). ^3^ It represents training the TICNET from scratch. ^4^ We used the pre-trained weights of Pascal VOC dataset to train the TICNET. ^5^ We used the pre-trained weights of Pascal VOC dataset for the transfer learning firstly and trained a model that can recognize common pointer surface defects, and then used the model for secondary transfer learning. CPD is the abbreviation for common pointer surface defects dataset.

**Table 5:** Comparative results for different detection algorithms.

Algorithm	mAP_0.5_	mAP_0.7_	ACC	DR
SSD	0.690	0.679	0.788	0.715
Retinanet	0.733	0.732	0.874	0.900
RFCN	0.824	0.813	0.875	0.903
Faster-RCNN	0.825	0.804	0.871	0.864
Cascade-RCNN	0.761	0.754	0.826	0.839
SSD *	0.615	0.566	0.647	0.643
RFCN *	0.738	0.731	0.801	0.828
Cascade-RCNN *	0.721	0.709	0.813	0.802
TICNET (Ours)	0.873	0.862	0.906	0.933

**Table 6:** The comparison statistics of algorithms on the different defects.

		Defects	Hot Stamping Paper Folds	Hot Stamping Paper Damage	Needle Leakage	Stains	Hair-Like Defects
	AVGConf						
Algorithm							
SSD			0.812	0.831	0.849	0.792	0.776
Faster-RCNN			0.925	0.920	0.942	0.944	0.957
TICNET (Ours)			0.969	0.957	0.974	0.971	0.989
